# Parallel functional annotation of cancer-associated missense mutations in histone methyltransferases

**DOI:** 10.1038/s41598-022-23229-2

**Published:** 2022-11-02

**Authors:** Ashley J. Canning, Susan Viggiano, Martin E. Fernandez-Zapico, Michael S. Cosgrove

**Affiliations:** 1 Department of Biochemistry and Molecular Biology, State University of New York (SUNY) Upstate Medical University, 4261 Weiskotten Hall, Syracuse, NY 13210 USA; 2 Schulze Center for Novel Therapeutics, Division of Oncology Research, Mayo Clinic, Rochester, MN USA

**Keywords:** Biochemistry, Enzymes, Structural biology, Cancer, Cancer genomics, Computational biology and bioinformatics, Data acquisition, Functional clustering, High-throughput screening, Machine learning, Predictive medicine, Protein analysis, Protein function predictions, Sequence annotation

## Abstract

Using exome sequencing for biomarker discovery and precision medicine requires connecting nucleotide-level variation with functional changes in encoded proteins. However, for functionally annotating the thousands of cancer-associated missense mutations, or variants of uncertain significance (VUS), purifying variant proteins for biochemical and functional analysis is cost-prohibitive and inefficient. We describe parallel functional annotation (PFA) of large numbers of VUS using small cultures and crude extracts in 96-well plates. Using members of a histone methyltransferase family, we demonstrate high-throughput structural and functional annotation of cancer-associated mutations. By combining functional annotation of paralogs, we discovered two phylogenetic and clustering parameters that improve the accuracy of sequence-based functional predictions to over 90%. Our results demonstrate the value of PFA for defining oncogenic/tumor suppressor functions of histone methyltransferases as well as enhancing the accuracy of sequence-based algorithms in predicting the effects of cancer-associated mutations.

## Introduction

Functional annotation of cancer-associated mutations is challenging^1,2^. Most missense mutations occur in positions with no known function, preventing identification of driver vs. neutral (passenger) mutations. Current functional annotation methods use nucleotide and amino acid (aa) sequence conservation to predict mutational pathogenicity^3–5^. Validation relies on mutant divergence in aa side chains compared to wild-type and statistically estimating the probability of positive selection relative to the background mutation rate^6^. However, changing a conserved aa does not always change function. Algorithms incorporating structural and thermodynamic information into functional predictions^7,8^ are limited by the paucity of structural information for protein conformational and liganded states. Predicting the impact of aa substitution on function is difficult for proteins in complexes. Predictions improve for well-characterized proteins, but such information requires costly, time-consuming protein purification and characterization. Knowing which mutations drive cancer is crucial for prioritizing cell- and animal-based studies, but functional prediction programs cannot reliably guide these high-cost experiments^6,9^.

We describe parallel functional annotation (PFA) for high-throughput characterization of cancer-associated missense variants of uncertain significance (VUS) without protein purification. We demonstrate the value of PFA with three Mixed Lineage Leukemia (MLL) family histone H3 lysine 4 (H3K4) methyltransferases that are among the most frequently mutated genes in cancer (Fig. S1A)^10–20^. Mutations in MLL family enzymes are associated with genome-wide aberrations in the patterns of H3K4 methylation, which are linked to abnormal transcriptional programs that promote malignancy^18,21–23^. Of hundreds of MLL1-3 VUS, most are at amino acid positions without known function (Fig. S1B). We screened 99 cancer-associated missense mutations in or around catalytic Suppressor of Variegation, Enhancer of Zeste, Trithorax (SET) domains, comparing results with two widely used functional prediction programs. Using functional annotation of three MLL paralogs, we discovered that combining two phylogenic and clustering parameters improved sequence-based functional prediction accuracy to > 90%. These results provide a foundation for improving computational methods to predict functional effects of cancer-associated mutations for biomarker discovery and precision medicine.

## Results

### Computational predictions of mutations

**Figure 1:**
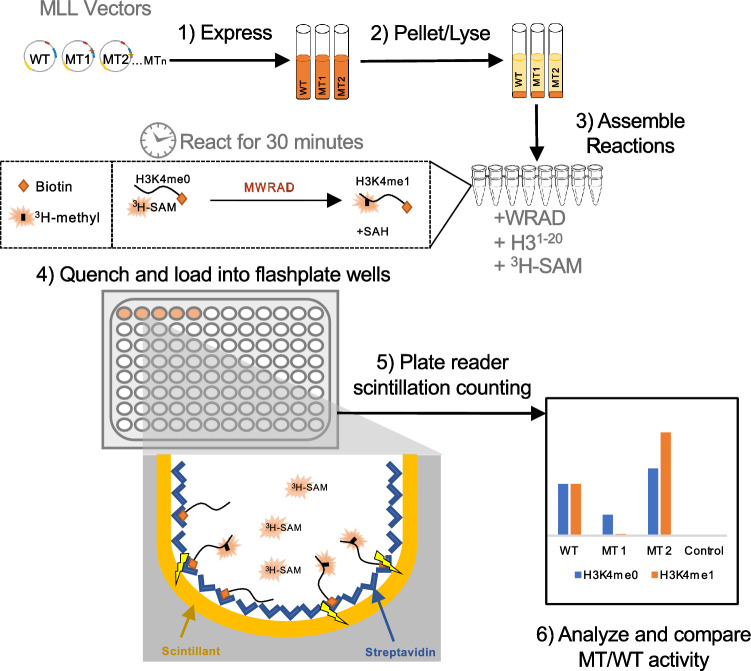
Workflow for the Parallel Functional Annotation (PFA) assay. (1) Recombinant expression plasmids for wild-type (WT) and mutants (MT) in *Escherichia coli* were induced in 5-ml cultures. (2) Culture pellets were lysed and clarified. Crude extract was normalized for equal amounts of recombinant protein using Coomassie-stained SDS-PAGE and/or western blotting. *E. coli* does not methylate histones, so substrates were not modified without recombinant protein and lysates were the enzyme source in assays. (3) Assays with lysates in PCR strip tubes were initiated with a temperature-equilibrated mixture containing subunits required for active histone methyltransferase complexes (WRAD), biotinylated histone H3 peptides (amino acids 1–20) as substrate, and radiolabeled S-adenosylmethionine (^3^H-SAM). (4) Reactions were transferred to commercially available streptavidin/scintillant-coated 96-well FlashPlates^34^ containing quenching reagent (step 4). Quenching times for end-point assays were determined using WT enzyme to ensure signal-to-noise within the linear range of the timecourse. (5) A plate reader detected signal for methylated biotinylated peptides captured by streptavidin near scintillant making removal of unincorporated ^3^H-SAM unnecessary. (6) Results were analyzed. H3K4me0, unmethylated H3; H3K4me1, monomethylated H3.

To better understand how well predictive tools categorize clinically relevant missense mutations in frequently mutated enzyme families, we functionally analyzed VUS in the catalytic SET domains of MLL1-3 (Fig. 1), comparing results with three widely used computational prediction programs. MLL enzymes catalyze histone H3 lysine 4 (H3K4) methylation^24^. Alterations are associated with genome-wide aberrations in methylation linked to malignancy. MLL1-3 are among the most commonly mutated genes in multiple cancers^25,26^. Of hundreds of MLL1-3 VUS, most are at amino acid positions without known function (Fig. S1).

**Figure 2:**
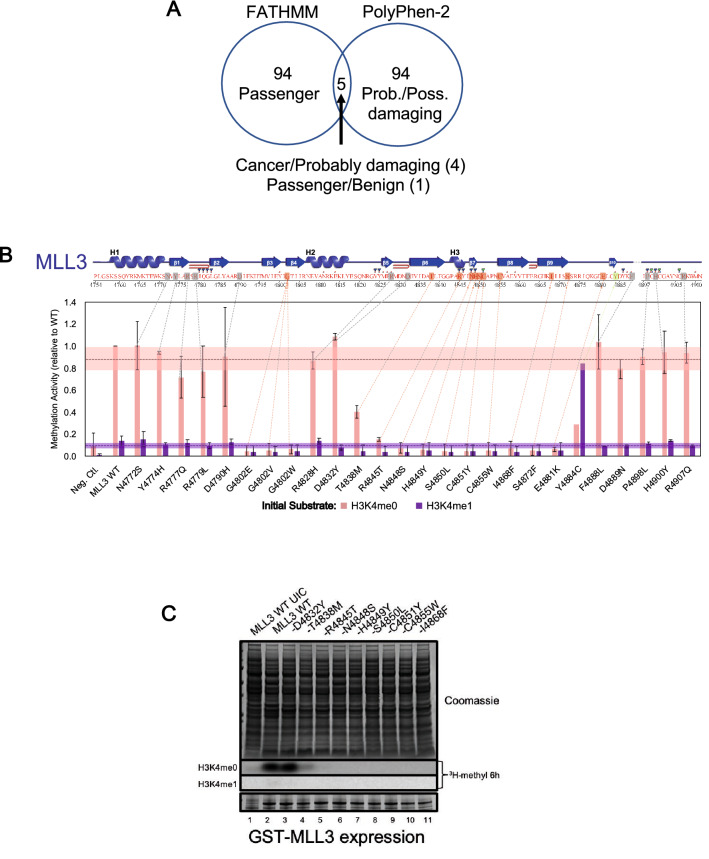
Parallel Functional Annotation (PFA) of cancer-associated histone methyltransferase variants of uncertain significance (VUS). (**A**) Venn diagram of FATHMM and PolyPhen-2 functional prediction of Mixed Lineage Leukemia (MLL) VUS: 5 of 99 mutations had overlapping predictions. (**B**) *Top,* catalytic SET domain secondary structure map from PDBsum based on PDB 5F59, amino acids 4754–4911. Shown are alpha helices (H1-3), beta sheets (β1-10) beta hairpin sturns (red coloured hairpin sturns), and ligand/metal binding residues: H3/SAH binding (red filled square), SAH binding (blue filled triangle), zinc ion binding (blue filled square/green filled triangle). *Bottom,* representative PFA of MLL3 VUS mutations by scintillation counting. Quenching was after 30 min with data normalized against WT. Pink and purple, assays initiated with H3K4me0, H3K4me1. Dashed lines and corresponding shaded regions, average and standard deviation (1σ), respectively, for all variants with activity > 50% of WT. Error bars, standard deviation from 2 independent experiments. (**C**) Representative results from PFA for MLL3 VUS mutations by fluorography of SDS-PAGE. *Upper*, Coomassie-stained gel of quenched enzymatic reactions; *middle*, signal from reactions with H3K4me0 (unmethylated) or H3K4me1 (monomethylated) peptides; *bottom*, expression of MLL3 variants by Coomassie-stained SDS-PAGE. Assays were as described for Fig. 1, limiting the recombinant subunits required for full enzymatic activity^31–33^ to minimize activity variation from differing MLL expression. Rates of monomethylation and dimethylation were determined using unmodified or monomethylated substrates. Activity depended on recombinant expression (no activity in uninduced control, UIC, lane 1). Lanes 2–11 show representative wild-type (WT) and variant MLL3 complexes, demonstrating that activity variation cannot be explained by differential expression. An uncropped version of Fig. 2C is shown in Fig. S11.

We compiled a list of 99 VUS in the last 260 aa of MLL1-3 within or near the catalytic SET domain, (29 in MLL1, 44 in MLL2, 26 in MLL3, Supplementary File 1) from the Catalog of Somatic Mutations In Cancer (COSMIC) database^27^ and from exome sequencing of 308 tumors of various origins at the Mayo Clinic^28^. We calculated functional impact scores for each mutation using the cancer-specific option of Functional Analysis Through Hidden Markov Models (v2.3) (FATHMM)^29^, the Polymorphism Phenotyping v2 (PolyPhen-2) functional prediction server, which incorporates structural information into annotations^7^, and CancerVar’s Oncogenic Prioritization by Artificial Intelligence (OPAI) server^30^. Using default disease thresholds, FATHMM predicted 4 mutations resulting in cancer; 96% were inferred as passenger mutations (Supplementary File 1**).** PolyPhen-2 scores suggested 89 mutations were probably damaging, 5 possibly damaging and 1 benign. The programs agreed on 1 benign/passenger and 4 cancer/probably damaging inferences (5%), but with high discordancy in functional inferences for the remaining ~ 95% of missense mutation positions (Fig. 2A)—the true functions of which are unknown. We also used CancerVar to predict the oncogenic potential of the 99 VUS (Supplementary File 1), and found that the majority (82%) have an uncertain probability of oncogenicity (OPAI score < 0.95). The disagreement among the programs led us to develop a high throughput functional assay to aid predictive tools in understanding the role of SET domain mutations in disease.

### PFA of mutations

To determine the true functional impact on enzymatic activity, we developed a cost-effective, high-throughput PFA platform for rapidly comparing enzymatic activity in variant and wild-type proteins. PFA involves parallel expression of wild-type and variant genes in small cultures (Fig. 1). We used characterization of all 99 VUS histone-methylation enzymes as a model, expressing wild-type or variant SET domains from recombinant plasmids in *Escherichia coli.* After cell lysis, assays were initiated by combining crude, normalized extracts with ^3^H-S-adenosylmethionine (^3^H-SAM), biotinylated histone peptide substrate, and cofactors or interacting proteins, in our case, purified recombinant subunits required for full enzymatic activity^31–33^. At specific timepoints, reactions were transferred to commercially available streptavidin/scintillant-coated 96-well FlashPlates^34^ containing quencher. A plate reader detected reaction signals, in our case for methylated biotinylated peptides captured by streptavidin proximal to scintillant. Data were normalized against wild-type enzyme activity (Fig. 2B and Figs. S2,S3,S4). All steps used 8-channel pipettors in a standard PCR thermocycler, allowing high-throughput parallelization.

**Figure 3:**
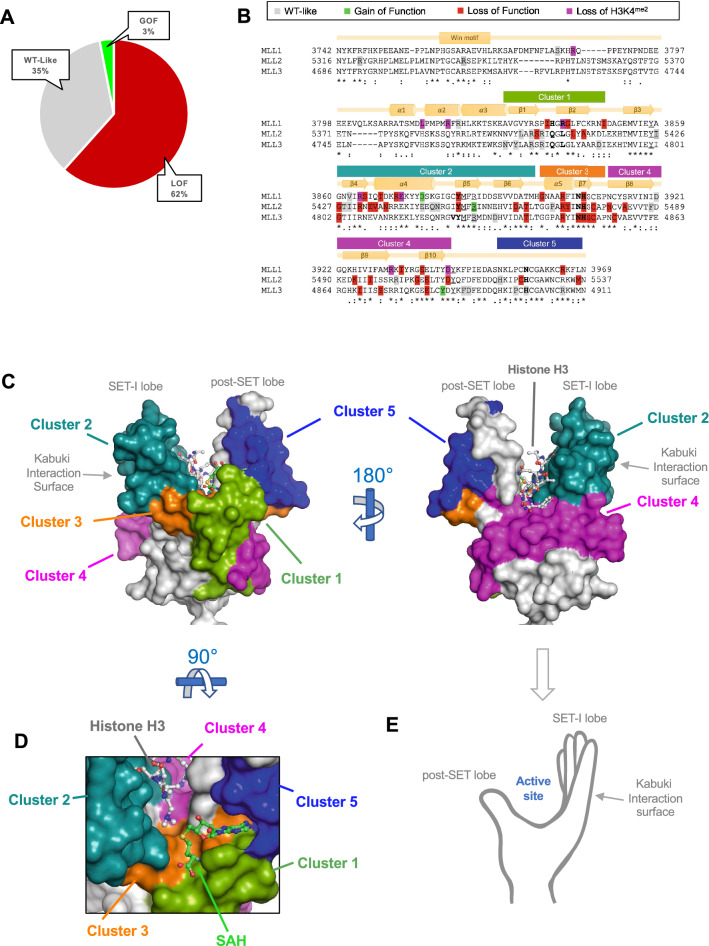
Structure–function annotation of methyltransferase cancer-associated variants of uncertain significance (VUS). (**A**) Proportions of neutral (wildtype [WT]-like), loss-of-function (LOF), and gain-of-function (GOF) variants from parallel functional annotation of 99 Mixed Lineage Leukemia (MLL) VUS. (**B**) Clustal Omega sequence alignment of the SET (active site-containing) domains of three MLL paralogs. Gray, neutral; green, GOF; red, LOF; pink, MLL1 mutations eliminating histone H3 lysine 4 (H3K4) dimethylation but not monomethylation. Annotation with PDBSum secondary structure is based on MLL1. Cluster 1–5 bars show putative missense mutation clusters. (**C**) Surface representation of the MLL1 SET domain (PDB code 2W5Z) showing mutation clusters, colored as in B). Cluster 2 LOF variants mapping to the nonactive-site surface of the SET-I lobe include mutations associated with human Kabuki syndrome when in MLL2^48–53,39,54,55^. Mutations impair complex assembly and enzymatic activity by disrupting a surface required for interaction with the RBBP5/Ash2L heterodimer necessary for catalysis^39^. Cluster 3 encompasses α-helix 5 through β-sheet 7 with the highly conserved “NHS” signature motif essential for SET activity^44,61–64^ in all 6 human MLLs^33^ near the fulcrum of the bilobed structure with direct contacts to S-adenosylmethionine at the coenzyme-binding pocket base. Cluster 4 LOF variants encompass residues in β-sheets 8–10 on a contiguous surface along the domain base. Mutations affect buried amino acids on the nonsolvent-exposed surface of β-sheets 8 and 9 (predicted to destabilize). One GOF-variant in MLL3 replaced tyrosine 4884, which inserts at the “Phe/Tyr switch” active site position and determines product specificity with cysteine^32,63,66–72^. This variant dimethylated but did not monomethylate, similar to a cancer-causing Y-to-C substitution in the polycomb SET domain EZH2 active site^71^. Cluster 5 encompasses the post-SET domain with the zinc-binding lobe (thumb) of the SET domain with 3 of 4 cysteines coordinating the zinc atom (fourth from cluster 3). Zinc is crucial for the adenine-proximal portion of the coenzyme-binding pocket. Gray text, SET-I and post-SET lobes (critical for methyltransferase activity) and Kabuki interaction surface; histone H3 and ball-and-stick model, active site position on the SET-I lobe. (**D**) Enlarged view of VUS mutation clusters converging on the active site with positions of substrate and co-factor product S-adenosylhomocysteine (SAH) indicated. (**E**) Positions of domain features.

To validate results, reactions were visualized by fluorography (Fig. 2C). Methylation activity depended on recombinant expression, with no activity in uninduced control (lane 1). Wild-type and variant MLL3 complexes demonstrated that activity variation was not explained by differing catalytic domain expression (lanes 2–11 and lower panel). Variation in enzymatic activity by fluorography qualitatively matched scintillation-counting results (Fig. S5). Furthermore, observed changes in relative activity for the subset of previously characterized mutations were consistent with the literature^12,13,32,33,35–39^, validating the assay. Of the 99 VUS characterized by PFA, 62% demonstrated loss-of-function (LOF) (activity < 50% of wild-type), 3% showed gain-of-function (GOF), and 35% showed no significant change (Fig. 3A).

### Biochemical consequences of VUS

To gain structural and biochemical insight into the variants, we used CLUSTAL-Omega sequence alignment^40^ annotated for aa conservation and secondary structure, and mapping onto X-ray structures of isolated SET domains (Fig. 3B,C, Figs. S2,S3,S4)^41–44^.

Most LOF variants clustered around five primary structural elements (Fig. 3B): Cluster 1 mapped to β-strands that, with an intervening loop, form part of the SAM-binding pocket at the “palm” of the SET domain (Fig. 3C,D,E). Mutations here likely alter β-sheet packing against the domain and disrupt the SAM-binding pocket. Positions of cluster 1 LOF variants had varying degrees of aa conservation among SET domains and in only 2 of 3 MLLs (Fig. 3B). Several neutral mutations, some in highly conserved positions, demonstrated that aa conservation was not always sufficient for functional predictions.

Cluster 2 encompassed residues between β-strands at a region thought to determine substrate specificity^45^ (Fig. 3B–E). Several LOF mutations mapped to opposite surfaces of the region (Figs. S2,S3,S4). LOF variants near the active site likely disrupted histone or cofactor binding. One mapped putative GOF variant showed increased dimethylation without changing monomethylation activity. The same position was mutated in another MLL without changing enzymatic activity (Fig. 3B, Figs. S2,S3). A different GOF variant increased dimethylation without changing monomethylation, mapping to a histone peptide-binding surface (Fig. S3). The same position was mutated in another MLL without changing activity (Fig. 2B, Fig. S4). Some cluster 2 LOF variants mapped to a nonactive-site surface where we demonstrated that mutations impair core complex assembly and enzymatic activity (Fig. 3C)^39^, an interaction confirmed by cryo-EM (Fig. S6)^55–57^. These observations emphasize the importance of incorporating functional information from multiple family members that may be different in their assembly with homologous subunits^33,38^.

Cluster 3 included a highly conserved NHS-motif essential for enzymatic activity^33,42,59–62^ that directly contacts SAM at the base of the coenzyme-binding pocket. High conservation and prior biochemical information likely accounted for correct functional inferences from FATHMM and PolyPhen-2 for NHS-motif mutations. However, these programs did not distinguish LOF and neutral mutations for the remaining 95% of variants, including cluster 1 and remaining cluster 3 variants that, based on structural information, are involved in forming the SAM/S-adenosylhomocysteine-binding pocket (Fig. 3D).

Cluster 4 LOF variants encompassed residues mapping to a contiguous surface along the SET domain base. LOF mutations in this cluster predominantly affected buried aa positions and were predicted to be destabilizing. One GOF variant was in a residue that inserts into the active site at a position that determines product specificity^32,33,61,64–70^. This variant showed a mixed phenotype of lost monomethyltransferase activity, but gained dimethyltransferase activity (Fig. 2B), similar to a cancer-causing substitution^71^ that is a lymphoma treatment target^72^.

Cluster 5 encompassed a domain that forms a zinc-binding lobe and provides 3 of 4 cysteine residues coordinating a zinc crucial to a portion of the coenzyme-binding pocket (Fig. 3D). LOF variants in this region likely destabilize the zinc-binding lobe, altering SAM binding.

### Comparing predicted and in vitro phenotypes

**Figure 4:**
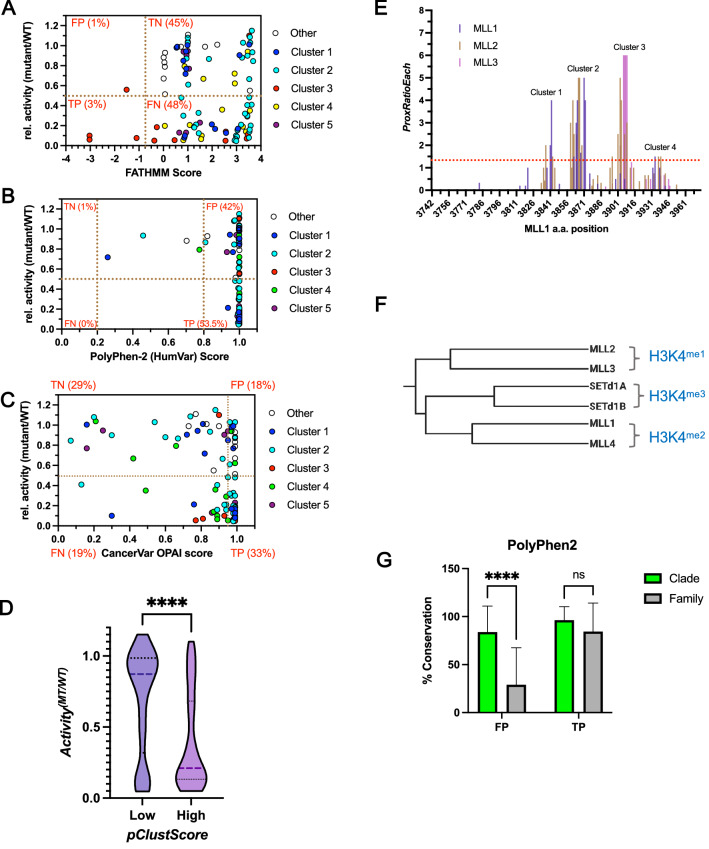
Comparison of predicted and in vitro phenotypes for cancer-associated missense variants of uncertain significance (VUS). (**A**) FATHMM scores vs. relative activity (mutant [MT]/wild-type [WT]) of VUS color coded by clusters. Clusters 1, 2, 4, and 5 have roughly equal density above and below 50% of wild-type activity (horizontal dotted line); vertical dotted line, FATHMM cancer default disease threshold ≤ − 0.75 (greater certainty that a mutation causes disease); white circles, 12 neutral mutations that did not fit in the 5 clusters; red dots, mutations corresponding to conserved “NHS” mutations in cluster 3 (motif essential for enzymatic activity). Three highly conserved cluster 3 residues were correctly called as true positives (TPs), but FATHMM lacked sensitivity to call the remaining cluster 3 loss-of-function variants despite similar activity loss. (**B**) PolyPhen-2 scores vs. relative activity of VUS. Vertical lines, PolyPhen-2 default disease thresholds: > 0.8 “probably damaging”, 0.2 to 0.8 “possibly damaging”, < 0.2 benign). (**C**) CancerVar OPAI scores vs. relative activity of VUS. Vertical line,default threshold (< 0.95) for variants with uncertain probability of oncogenicity. (**D**) Violin plot of mean activity differences between VUS with low (< 1.5) or high (> 1.5) parallel cluster scores (*pClustScore*). Significance was from 2-tailed unpaired t-tests. Dashed line, median; dotted lines, upper and lower quartiles. (**E**) Variant *ProxRatioEach* scores showing proximity of adjacent missense mutations in each protein, plotted as a function of amino acid position using Mixed Lineage Leukemia (MLL) 1 numbering. (**F**) Clustal Omega phylogenetic cluster analysis of human SET1/MLL proteins shows three clades diverged in product specificity (me1, 2, 3 is degree of methylation)^33^. (**G**) Comparison of family vs. versus clade conservation scores in PolyPhen-2 false-positive (FP) and true-positive (TP) amino acid positions. Two-way ANOVA compared means within groups. ****P < 0.0001; ns, P > 0.05.

To determine how well functional annotation programs predict biochemical changes in MLL family VUS, we plotted FATHMM, PolyPhen-2 and CancerVar scores against methyltransferase activity normalized to wild-type (Fig. 4A–C). FATHMM scores clustered into three regions (Fig. 4A). Of 99 missense mutations, 3 representing true-positive (TP) predictions had activity < 50% of wild-type with FATHMM scores meeting the default disease threshold (≤ − 0.75)^29^. Another cluster 3 prediction fell into the false-positive (FP) region, with tenuous assignment because activity was barely above the 50% threshold. Another region representing true-negative (TN) predictions, containing 45% of mutations, had activity > 50% of wild-type with FATHMM scores > − 0.75. The third region representing false-negative (FN) predictions (48% of mutations) had activity < 50% wild-type and FATHMM scores indicating no disease.

In further cluster classification, FATHMM correctly called all 12 neutral mutations not within the five cluster groupings. FATHMM correctly predicted functional impacts of only 6% of all 51 LOF mutations, with FN inferences for 94%. FATHMM had mixed results for variants within the structural clusters. Three highly conserved cluster 3 residues were correctly called as TPs; FATHMM lacked sensitivity to call the remaining LOF variants despite similar activity loss (Fig. 4A).

PolyPhen-2 clustered the 99 missense mutations predominantly into two groups (Fig. 4B): 95% had scores > 0.8, predicting “probably damaging.” Mutations with activity < 50% of wild-type (53.5% of total) represented TP predictions. All but 4 of the remaining (42% of total) with activity > 50% of wild-type represent FP predictions. PoylPhen-2 incorporated structural information into the predictions^7^, but in contrast to FATHMM, lacked precision to adequately distinguish FP from TN inferences.

CancerVar clustered missense mutation into 4 regions (Fig. 4C): 53% had OPAI scores ≥ 0.95 and were predicted to be oncogenic. Mutations with activity < 50% of wild-type represented TP (33%) and FN (19%) predictions, whereas mutations with activity > 50% of wild-type represent FP (18%) and TN (29%) predictions.

Together, while the programs show general agreement for the few mutations in amino acid positions with prior functional information, they struggled to correct classify the impact of the remaining mutations- despite incorporating structural information into the prediction. These results reinforce the need for additional high throughput biochemical annotation methods to identify the variables that are most important for accurate functional predictions.

### Predicting impact of mutations

The contradictory FATHMM, PolyPhen-2 and CancerVar results underscore the difficulties of inferring the functional impact of VUS using prediction programs that rely primarily on aa sequence conservation. To identify the most important variables for predicting functional impact on MLL enzymes, Supplementary File 2 has 14 potential explanatory parameters including changes in aa physical–chemical properties: number of side chain atoms (*ΔAtoms*) or hydrogen bond donors or acceptors, charge, hydrophobicity, side chain volume, and the predicted changes in unfolding free energy (*ΔΔG*) upon point mutation. Substitution probabilities were from the *BLOSUM62* matrix^73^.

We tested inclusion of additional variables inferred from functional annotation observations to improve predictions. LOF mutations clustered nonrandomly in specific structural regions, suggesting that clustering might indicate altered function. We calculated a missense-mutation “parallel-cluster score” (*pClustScore*) from the proximity of adjacent missense mutations within each protein *(ProxScoreEach*) and the proximity of the aggregate of all missense mutations from MLL family members projected onto a single aa sequence (*ProxRatioAll*). The average enzymatic activity was significantly lower for missense mutations with high vs. low cluster scores (P = 0.0001) (Fig. 4D). Missense mutations clustered into 4 groups corresponding with the structural analysis; the fifth group (post-SET domain) showed some clustering (Fig. 4E, Fig. S7)*.* Differences in distributions of missense mutations among family members suggested a subgroup of missense mutations had differential effects on each protein.

To understand reasons for the large number of PolyPhen-2 FP inferences, we studied differences in aa conservation scores comparing alignments of all SET1 family SET domains with members of each phylogenetic subfamily (clade). Comparison of the six human SET1/MLL family members showed three clades that diverged in target gene and product specificity (number of H3K4 methylations) (Fig. 4F)^33^. PolyPhen-2 TP predictions showed little difference in average family vs. clade conservation scores. FP predictions had family-conservation scores significantly lower than clade-conservation scores (P < 0.0001) (Fig. 4G), indicating that despite high conservation among orthologs, positions that differed among paralogs had diminished predicted importance. To test if including phylogenetic information improved functional predictions, we used *Mutation Assesso*r^74^ to compute functional impact scores (*FI-Score*) for each missense mutation. *FI-Score* is derived from a combinatorial entropy approach that simultaneously computes a “family conservation” score (*VC-Score*) and “specificity score” (*VS-Score*) based on conservation among orthologs within each subclade^74,75^.

**Figure 5:**
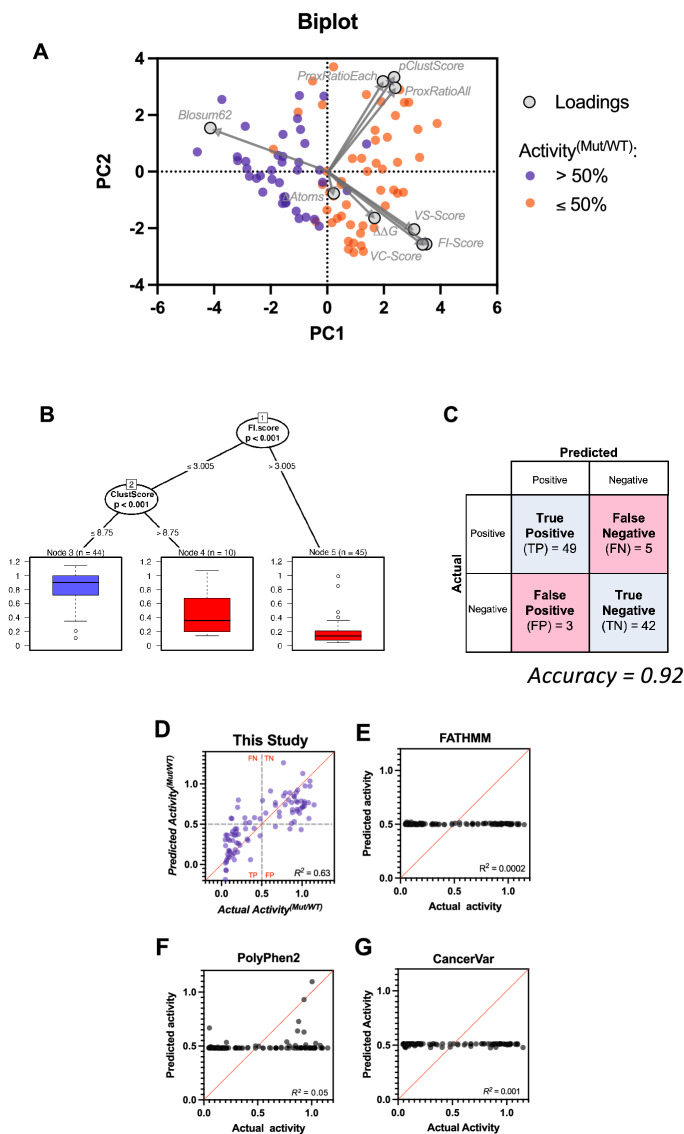
Phylogenetic and clustering parameters predict functional impact of cancer-associated missense variants of uncertain significance (VUS). (**A**) Principal component (PC) biplot of significant phylogenetic (*FI-Score, VC-Score and VS-Score*), clustering (*pClustScore, ProxRatioAll, ProxRatioEach*) and physical–chemical (*ΔAtoms, Blosum62, ΔΔG*) parameters. Red, VUS with enzymatic activity ≤ 50% of wild-type (WT); blue, VUS with activity > 50% of WT. Mut, mutant. (**B**) Recursive partitioning classification tree for enzymatic activity using *FI-score*, *pClustScore*, *ΔAtoms, Blosum62 and ΔΔG* parameters for MLL1-3 VUS. Circles, internal nodes that can be partitioned into subnodes; boxes, terminal nodes; red, VUS with activity ≤ 50% of WT; blue, VUS with activity > 50% of WT. Circles, P values input nodes; box plots of *Activity*^*(MT/WT)*^ values are in terminal nodes. (Goodness of Fit R^2^ = 0.65, RMSE = 0.22) (**C**) Confusion matrix showing predictive accuracy of the tree based on the tenfold cross-validation scheme. The recursive partitioning algorithm was repeated^85^ with 10 rounds of fitting, each using randomly chosen data subsets, with 90% training set and 10% testing set. **D-G**) Actual vs. Predicted plots. X-axes, actual activity; y-axes, predicted activity based on the regression model. Red diagonal line, line of identity; dashed lines, cutoff for VUS with less than or greater than 50% WT activity. (**D**) *FI-Score* and *pClustScore* parameters as predictors. (**E**) FATHMM inference score as predictor. (**F**) PolyPhen-2 inference score as predictor. (**G**) CancerVar Oncogenic Prioritization by Artificial Intelligence (OPAI) score as predictor. Shown are adjusted *R*^*2*^ values.

Clustering parameters (*ProxRatioEach, ProxRatioAll, pClustScore*) and phylogenetic parameters (*FI-,VC-,and VS-scores*) each demonstrated statistically significant relationships with mutant activity relative to wild-type, *Activity*^*(Mut/WT)*^ (Spearman’s P $\le$ 0.01). *ΔAtoms*, *ΔΔG*, and *BLOSUM62* parameters demonstrated weak but significant associations with *Activity*^*(Mut/WT)*^ (Spearman’s P = 0.04); other physical–chemical parameters were not significantly correlated (Fig. S8). Contributions of variables to *Activity*^*(Mut/WT)*^ were determined by principal components regression on 14 parameters. Consistent with correlations, phylogenetic and clustering parameters and *BLOSUM62, ΔΔG* and *ΔAtoms* were major contributors to variation in methylation rates (Fig. S9). Using only these covariates identified three principal components that collectively accounted for ~ 76% of variation (R^2^ = 0.61, P < 0.0001 when regressed on *Activity*^*(Mut/WT)*^, Fig. S10). Phylogenetic parameters accounted for the largest proportion of observed data variation (37%); clustering parameters contributed most strongly to PC2 (27%), and *ΔAtoms* to PC3 (12%). PC1 vs. PC2 scores revealed groupings separated between low and high enzymatic activity along PC1 (Fig. 5A), suggesting that phylogenetic and clustering parameters were most predictive of methylation rates. *FI-Score* was strongly associated with *VC-Score*, *VS-Score* (Spearman’s P < 0.0001) and was the phylogenetic parameter for further analyses. Because of strong association between *pClustScore* and *ProxRatioAll*, and *ProxRatioEach* (Spearman’s P < 0.0001), *pClustScore* represented clustering parameters (Fig. S8).

A regression tree using an unbiased recursive partitioning algorithm^76^ showed how phylogenetic, clustering, and physical parameters influenced methylation variability among missense mutations. The first breakpoint for distinguishing variants with high vs. low activity was based on *FI-Score*, a measure of conservation and phylogenetic differences among paralogs (Fig. 5B). Almost all VUS variants with *FI-Scores* > 3.005 were correctly classified as LOF with very low activity (P < 0.001). For VUS variants with *FI-Scores* ≤ 3.005, *pClustScore* became the major factor distinguishing high- vs. low-activity variants. *Blosum62*, *ΔAtoms* and *ΔΔG* parameters were not significant. Thus, combining *FI-Score* and *pClustScore* was significantly better at predicting the functional impact of VUS mutations (R^2^ = 0.63) than FATHMM (R^2^ = 0.0002), PolyPhen-2 (R^2^ = 0.05) or CancerVar (R^2^ = 0.001) (Fig. 5D–G).

To test the predictive power of these two parameters, we repeated the recursive partitioning algorithm using tenfold crossvalidation^77^. *FI-Score* and *pClustScore* correctly predicted the functional impact of ~ 92% of VUS variants (Fig. 5C, Table S1), (compared to 51% FATHMM, 55% PolyPhen-2, 62% CancerVar Table S2). Thus, functional impact predictions were significantly improved by combining aa conservation information on all related proteins plus conservation of key positions among orthologs that differentiate unique functions of paralogs, with clustering density of missense mutations that define functional areas of protein folds.

## Discussion

We describe the rapid, economical PFA method for functionally annotating VUS without enzyme purification, modeled using histone-modification enzymes. Collecting functional information on 99 mutations took 1–2 weeks of benchwork. Results for a subgroup of missense mutations were similar to previous characterizations, validating PFA. Contrary to prediction algorithms, we found that 62% of VUS mutations result in loss of histone methyltransferase activity while 35% showed no observable defects, suggesting they are passenger mutations or they disrupt an activity not present in the assay. Of VUS mutations, 3% led to an observable gain- or switch-of-function, including one with alterations in product specificity similar to those observed in a SET domain of EZH2, which is currently being targeted by therapeutics as a lymphoma treatment^71,72,78–80^. In addition, we identified new LOF and GOF variants in uncharacterized aa positions.

PFA is most useful for parallel screening of large numbers of missense mutations for altered enzymatic function. PFA is easily modified to screen mutations in nonenzymatic subunits as long as they are required for enzymatic activity, for estimating preliminary kinetic parameters (e.g., *V*_*max*_), and screening for variants sensitive to inhibiting or enhancing compounds. Other coupled fluorescence-based assays that measure formation of S-adenosyl-homocysteine^34^ require purified enzyme to reduce off-target methylation or fluorescence quenching. PFA uses crude extracts, biotinylated substrates, and FlashPlates, eliminating steps to purify protein and remove unincorporated ^3^H-SAM before measurement. PFA can be used for other histone-modification enzymes, if functionally expressed in *E. coli*.

Drawbacks include the lack of posttranslational modifications, if required for activity. The assay likely misses mutations that do not alter enzymatic activity but affect GOF interactions with proteins or nucleic acids absent from the assay. Nonetheless, PFA produced insights about sequence-based computational predictions and suggested mechanisms for VUS contributions to cancer.

By combining functional annotation of three paralogs, we discovered sequence-based phylogenic and clustering parameters that dramatically improved functional predictions over three computational prediction programs. We noticed that most Polyphen-2 FP mutation positions were conserved among orthologs, but not among paralogs. Computational programs that ignore these phylogenetic differences likely diminish the importance of aa positions that are highly conserved within a phylogenetic subclade, but differ between subclades that diverged for specific functions. Our observation that the six human SET1/MLL family members fall into three phylogenetic subclades that diverged in product specificity (Fig. 4E)^33^ may explain why FATHMM, PolyPhen-2 and CancerVar programs struggled to predict the functional impact of MLL VUS **(**Table S2**)**.

The importance of phylogenetic information in functional prediction was recognized in a combinatorial entropy approach with *Mutation Assessor*^74,75^ that provides a missense-mutation *FI*-*Score* based on the overall conservation of an aa position and conservation of “specificity residues” that differ among paralogs^74^. For PFA, *FI-Score* explained the largest proportion of variation in methylation rates among MLL mutations (36%, Fig. S10), a significant improvement over physical–chemical and BLOSUM62 parameters combined, which explained < 10% of methylation rate variation. *FI-score* was necessary but not sufficient for the best functional predictions.

Missense-mutation proximity analysis has identified potential driver genes based on clustering in functional domains—indicating positive selection. Several approaches use sequenced-based or structure-based approaches to quantitively identify missense mutation clusters in oncogenes^81–83^. Given the paucity of structural information for most proteins, sequence-based parameters are desirable. Sequence-based clustering algorithms predominantly focus on identifying potential oncogenes, but can be useful for identifying structural features required for function. We found that most LOF mutations clustered around at least four unique structural elements involved in substrate or cofactor binding, or complex assembly. We noticed differences in clustering patterns among paralogs that may reflect differences in inactivation mechanisms. Based on the *Mutation Assessor* analogy, we computed a VUS clustering score accounting for these differences. The *pClustScore* parameter was better at predicting methylation rates than *ProxRatioAll* and/or *ProxRatioEach* parameters (Table S3), demonstrating complementarity.

Combining *FI-Score* with *pClustScore* described ~ 70% of methylation rate variability, without contributions from physical–chemical parameters often used in prediction algorithms. This level of variability was sufficient to predict functional impacts of VUS with up to ~ 90% accuracy. These results suggest that phylogenetic and clustering parameters from parallel analysis of family members provided important constraints for accurately modeling the functional impact of VUS mutations, particularly for families with multiple paralogs.

This work demonstrates how increasing knowledge of the impact of missense mutations on protein structure and biochemistry improves overall functional annotations. Application of similar high-throughput methods with other proteins will help identify all parameters required for accurate, broadly applicable, sequence-based functional predictions of missense mutations associated with disease.

## Methods

### Mutagenesis

pGST expression plasmids encoding the C-terminal 260 aa of each wild-type MLL family member were used as templates^33^. MLL family constructs consisted of residues MLL1 (3745–3969) (KMT2A, UniprotKB ID Q03164); MLL2 (also known as MLL4) (5319–5537) (KMT2B(D), (UniprotKB ID O14686); and MLL3 (4689–4911) (KMT2C, UniprotKB ID Q8NEZ4). Site directed mutagenesis was performed using QuickChange II XL kit (Agilent). In-house Sanger sequencing was used to confirm the presence of the intended sequence variant and the absence of unintended mutations.

### Protein expression and lysis

Colonies from transformed *E. coli* cells (Rosetta II (DE3) pLysS, Novagen) were used to inoculate 5 ml TBII media with 50 µg/ml carbenicillin and 25 µg/ml chloramphenicol and cultures were grown overnight with shaking at 30 °C. For PFA, 0.1 ml overnight culture was added to 5 ml fresh TBII with 50 µg/ml carbenicillin and 25 µg/ml chloramphenicol and grown at 37 °C with shaking at 200 rpm to OD600 ~ 1.0. Cultures were chilled on ice for 30 min, induced with 1 mM IPTG, and shaken at 200 rpm for 24 h at 16 °C. Cells were harvested by centrifugation at 4,000 rpm at 4 °C and pellets were resuspended in lysis buffer (50 mM Tris pH 7.5, 1 mM TCEP, 300 mM NaCl, 1 µM ZnCl_2_) supplemented with a complete protease inhibitor EDTA-free tablet (Roche Applied Science), 1XBugBuster (Novagen), and 0.25 mg/ml DNase A. Resuspended pellets were incubated at 4 °C with gentle rotation for 3 h. Cell lysates were harvested by centrifugation at 20,000 RPM at 4 °C. Supernatant was collected and pellets were discarded. Lysates were aliquoted, flash frozen and stored at − 80 °C. The expression level of each mutant was determined by 4–15% SDS PAGE using Mini-PROTEAN TGX gels (Bio-Rad) and Coomassie staining. Imaging and densitometry used a Bio-Rad Chemidoc Imager. Expression and purification of MLL core complex subunits WDR5, RbBP5, Ash2L and DPY-30 were as previously described^33^.

### Parallel functional annotation assays

Unmodified and H3K4 monomethylated histone H3 peptides (residues 1–20) tagged with GGK-biotin and C-terminal amidation were synthesized by GenScript and purified to > 95% purity. For methyltransferase assays, an equal volume of wild-type or mutant lysate was incubated with 3 µM WRAD, 250 µM H3 peptide (unmodified or monomethylated), and 1–2 µCi [^3^H]-SAM (PerkinElmer Life Sciences) in assay buffer (20 mM Tris pH 8.5, 1 mM TCEP, 200 mM NaCl, 1 µM ZnCl_2_). Samples were incubated at 15 °C for 30 min. Lysates from cells transformed with empty vector (pGST II) or uninduced wild-type plasmids served as negative controls. Reactions were quenched with 0.5 M EDTA (1:1, v:v). Quenched reactions were brought to 200 µL using assay buffer with 0.5 M EDTA and 0.2 mg/ml BSA and transferred to 96-well streptavidin-coated FlashPlate microplates (PerkinElmer). Samples were incubated overnight at 4 °C to allow binding of biotinylated H3 peptide to the streptavidin-coated surface before scintillation counting in a Hidex Sense Plus microplate reader (LabLogic). For the gel-based fluorography assays, reactions were quenched with SDS-loading buffer and separated by 4–12% BisTris SDS-PAGE (LifeTechnologies) at 200 V for 30 min. Gels were stained with Coomassie, imaged, then placed in enhancing solution (Enlightening, PerkinElmer Life Sciences) for 30 min at room temperature. Gels were dried for 2.5 h at 72 °C under constant vacuum and exposed to film (Eastman Kodak Co. Biomax MS Film) at − 80 °C for 6–72 h before developing. Densitometry using ChemiDoc ImageLab (BioRad) software was used to quantify H3 peptide methylation.

### Missense mutation clustering analysis

*pClustScore* was derived from the sum of two proximity parameters calculated using a modification of the approach in Tamborero et al.^81^. Missense mutation proximity parameters were calculated by counting the number of missense mutations in a window + /− 7 aa around each mutation, then dividing by the distance to the nearest missense mutation. The window for mutations was chosen based on an analysis showing that 25% of all neighboring mutations in the Catalogue of Somatic Mutations in Cancer (COSMIC) database are within 7 aa of each other, which represents the first quartile of nearest-neighbor distances^83^. *ProxRatioEach* was calculated based on the proximity of missense mutations within each protein and *ProxRatioAll* was calculated for the combined mutations for all three family members projected onto a single sequence. *ProxRatioAll* and *ProxRatioEach* were correlated (Fig. S8), indicating they provide complementary measures of mutational proximity. We therefore summed the two values to derive the single clustering parameter *pClustScore*. Multiple regression analysis showed that *pClustScore* was more accurate at predicting H3K4 methylation rates than either parameter alone, or when both parameters were used without summation (Table S3).

### Statistical methods

Principal components regression analysis was performed with GraphPad Prism version 9.3.0 for MacOS (GraphPad Software, San Diego, California USA). Component selection was based on the principal components with the largest eigenvalues that together explained at least 75% of the total variance. Recursive partitioning tree regression was performed using the R-based web-implementation of the R package as described^76,84^. Model validation was performed using tenfold crossvalidation with quantile categorization^76^, using 90% of the data as the training set and 10% to test the model. Validation was repeated 10 times using a different randomly chosen training and test set.

## Supplementary Information

Supplementary Information 1.

Supplementary Information 2.

Supplementary Information 3.

## Data Availability

The data generated during and/or analyzed during the current study are included in this published article (and its Supplementary Information files).
