# Supplementary material for: Parallel functional annotation of cancer-associated missense mutations in histone methyltransferases
Source: Sci Rep. 2022 Nov 2;12:18487. doi: 10.1038/s41598-022-23229-2 (PMC9630446; doi:10.1038/s41598-022-23229-2)
Supplement: Supplementary file 3 — Supplementary Information 3. [file 41598_2022_23229_MOESM3_ESM.docx]

# EXTENDED DATA FIGURES

**Parallel functional annotation of cancer-associated missense mutations in histone methyltransferases**

**Ashley J. Canning^1^, Susan Viggiano^1^, Martin E. Fernandez-Zapico^2^, and Michael S. Cosgrove*^1^**

*^1^State University of New York (SUNY) Upstate Medical University, Department of Biochemistry and Molecular Biology, Syracuse, New York.*

*^2^ Schulze Center for Novel Therapeutics, Division of Oncology Research, Mayo Clinic, Rochester, Minnesota.*

***Keywords:*** Histone modification, methylation, posttranslational modification, functional annotation, cancer associated missense mutations, Variants of Uncertain Significance (VUS), sequence-based functional prediction, Parallel Functional Annotation (PFA), High-throughput assay, precision medicine, Principal component regression, regression tree, recursive partitioning, FATHMM, PolyPhen-2, Mutation Assessor, CancerVar, oncogenic mechanism

*Corresponding Author:

Michael S. Cosgrove, Ph.D. Department of Biochemistry and Molecular Biology, SUNY Upstate Medical University, 4261 Weiskotten Hall, Syracuse, New York 13210, USA. Phone: (315) 464-7751, Email: cosgrovm@upstate.edu

**A**

**Fig. S1**


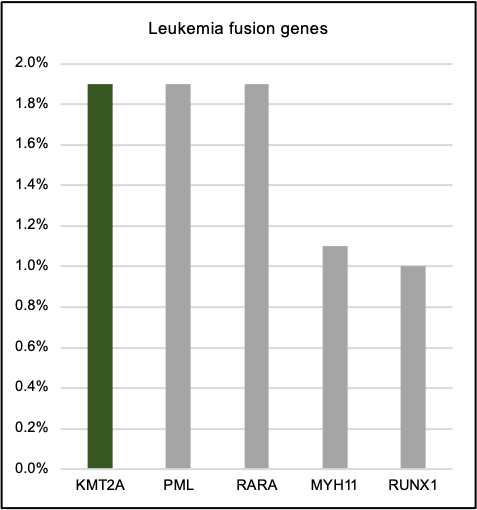

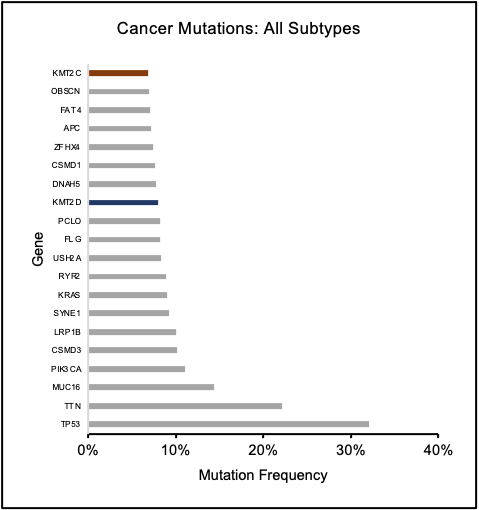


**Figure S1**: Histone-lysine N-methyltransferase subclass 2 (KMT2)/Mixed Lineage Leukemia (MLL) proteins are frequently mutated in cancers. (A) From a nonredundant collection of patient-derived sample sets (cBioPortal.org), the top 20 most frequently mutated genes are shown for all subtypes (left panel), with the top 5 most frequent fusion genes in all leukemias (right panel). (B) Lollipop models of all missense, fusion, and truncating mutations recorded for MLL1 (top), MLL2 (middle), and MLL3 (bottom) genes (cBioPortal nonredundant sample set, as of March 2021). According to the cBioPortal for Cancer Genomics (32), MLL1, MLL2, and MLL3 are among the most commonly mutated genes across multiple cancer subtypes, with MLL2 (KMT2D) and MLL3 (KMT2C) ranking among the top 20 most frequently mutated genes in all subtypes. MLL1 (KMT2A) is the most frequently mutated gene in infant acute leukemias, with MLL1 fusions present in up to 70% of infants with acute lymphocytic leukemia (33). While hundreds of cancer-associated missense mutations have been found in the open reading frames of MLL1-3, most occur in amino acid positions with no known function.

**B**


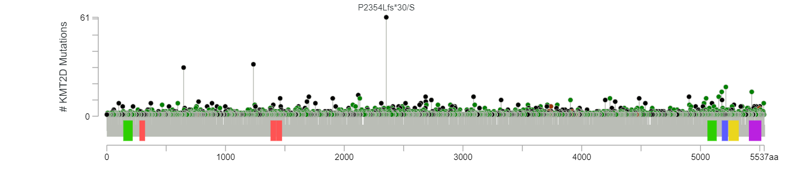

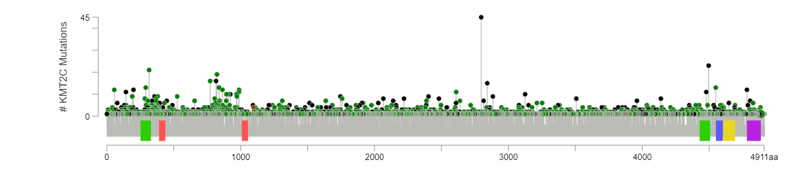

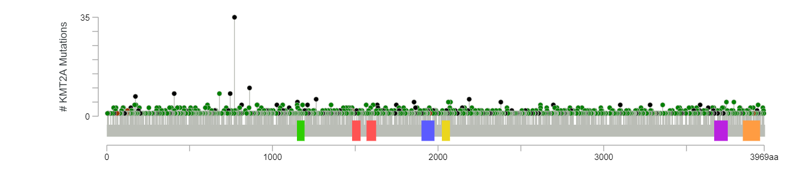


**MLL1**

**MLL2**

**MLL3**


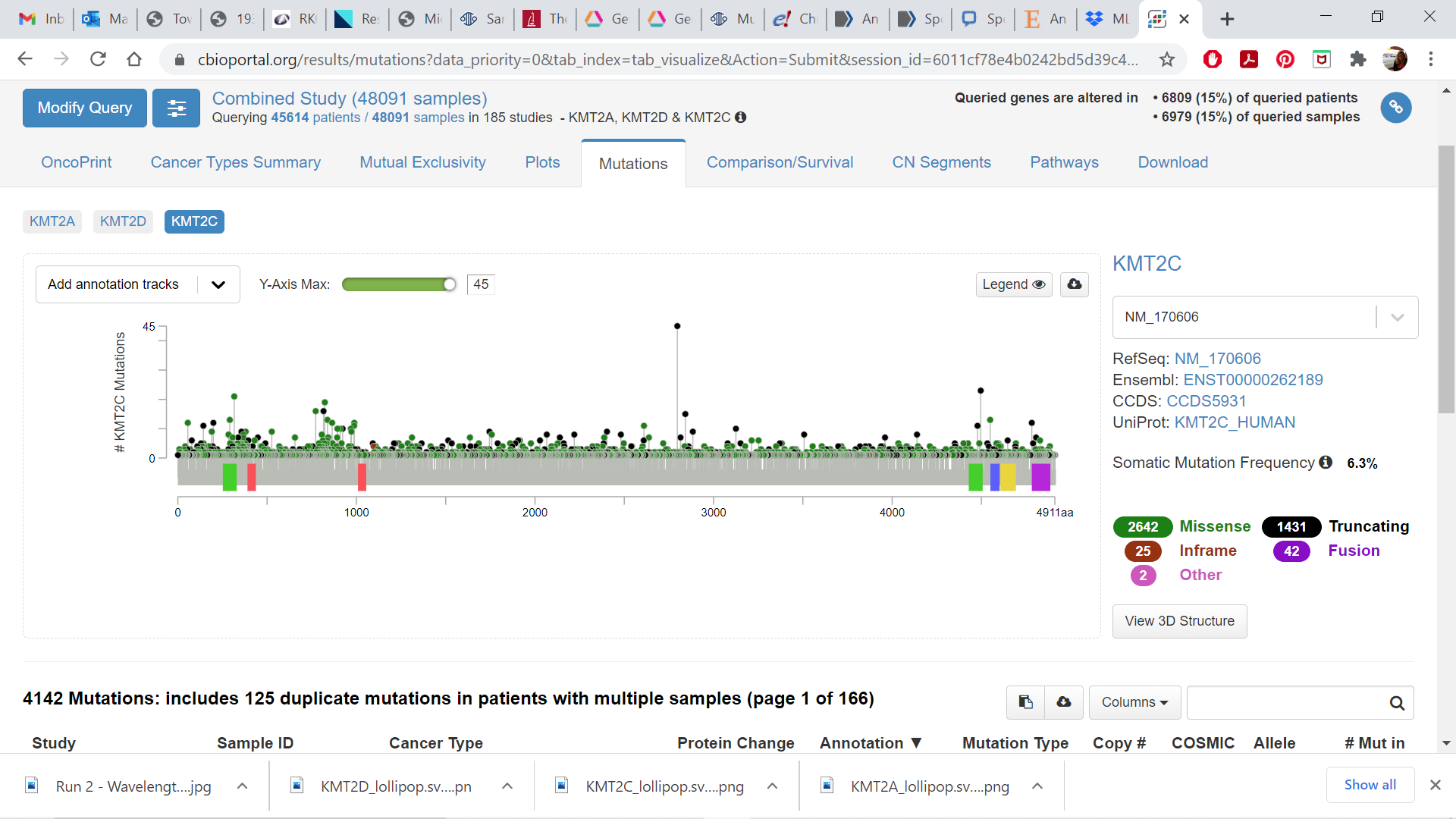

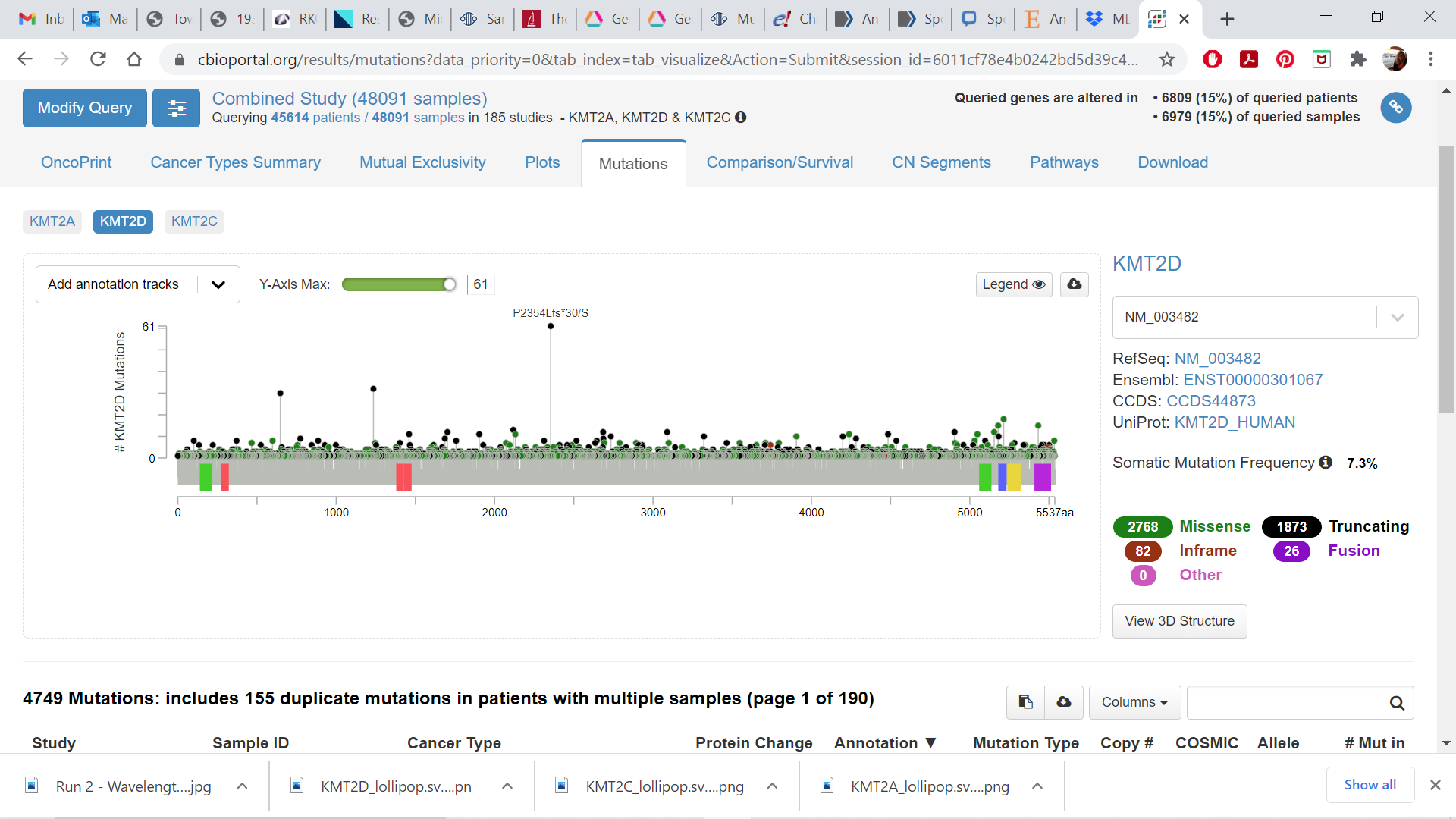

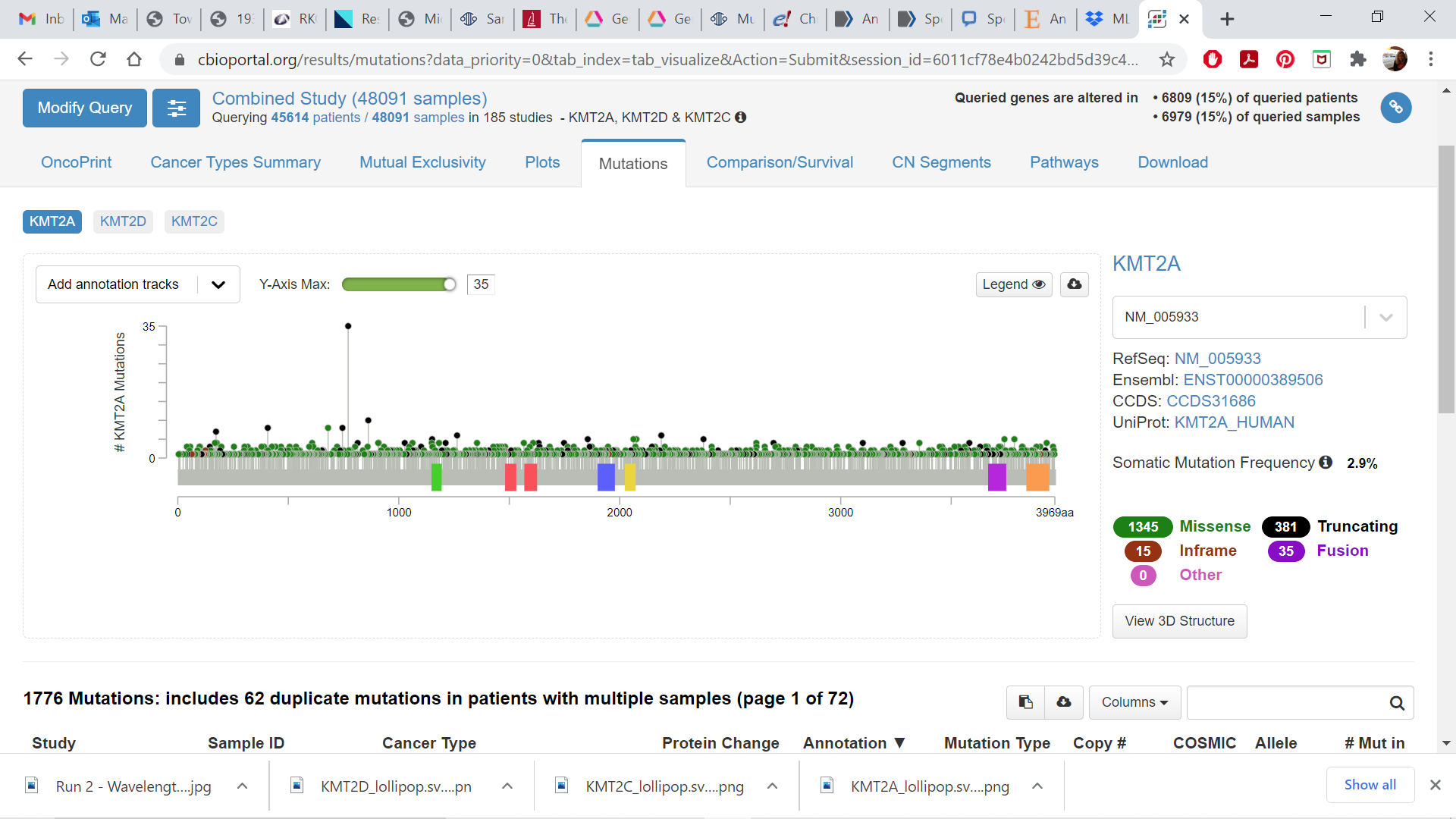


**Fig. S2**

**Figure S2:** Parallel functional annotation assay results of MLL1 cancer-associated variants of uncertain significance (VUS) mutations. (A) Top: MLL1 catalytic SET domain secondary structure map generated by PDBsum based on MLL1_SET_ structure (PDB 2W5Z, aa 3808-3969). Alpha helices (H1-3), beta sheets (β1-10) and respective beta hairpin turns (⊃) are annotated, and ligand/metal binding residues are labeled as follows: H3/SAH binding (▪️), SAH binding(▲), zinc ion binding (▪️/▲) for MLL1 secondary structure and primary sequence of MLL1 catalytic SET domain residues 3808-3969. Color coding: loss of mono- and dimethyltransferase activity (red), loss of only dimethylation activity (pink), gain of methyltransferase activity (green) or no change to catalytic activity (gray). Bottom, histogram showing results for assays initiated with H3K4me0 (unmethylated) and H3K4me1 (monomethylated) peptides, which are green and dark green bars, respectively. Horizontal dashed lines represent the average for all variants with activity >50% of wild-type. Results are shown for one experiment. Increased dimethylation activity is seen for gain-of-function mutant D3876N; substitution of the homologous residue in MLL2 does not result in increased dimethylation activity (see E5444K in Fig. S3A). B) Results of MLL1 cancer-associated missense mutations mapped onto the 3-dimensional structure of the MLL1 SET domain (PDB code: 2W5Z) (3) color coded as in (A).

**Figure S3:** Parallel functional annotation assay results of MLL2 cancer-associated missense (CAM) mutations. (A) Top: MLL2 catalytic SET domain secondary structure map generated by PDBsum based on MLL2_SET_ structure (PDB 4Z4P, aa 5380-5540). Alpha helices (H1-3), beta sheets (β1-12) and respective beta hairpin turns (⊃) are annotated, and ligand/metal binding residues are labeled as follows: SAH binding(▪️/▲) and zinc ion binding (▪️/▲). Color coding: loss of mono- and dimethyltransferase activity (red), loss of only dimethylation activity (pink), gain of methyltransferase activity (green) or no change to catalytic activity (gray). Bottom, histogram showing results for assays initiated with H3K4me0 (unmethylated) and H3K4me1 (monomethylated) peptides, which are pale blue and orange bars, respectively. Horizontal dashed lines and corresponding shaded regions represent the average and one standard deviation (1σ), respectively, for all variants with activity greater than 50% of wild-type. Error bars represent the standard deviation from two independent experiments. Increased dimethylation activity is seen for gain-of-function mutant R5454Q; substitution of the homologous residue in MLL3 does not result in increased dimethylation activity (see R4828H in Fig. S4A). B) Results of MLL1 CAM mutations mapped onto the three-dimensional structure of the MLL2 SET domain (PDB code: *4Z4P*) (2) color coded as in (A).


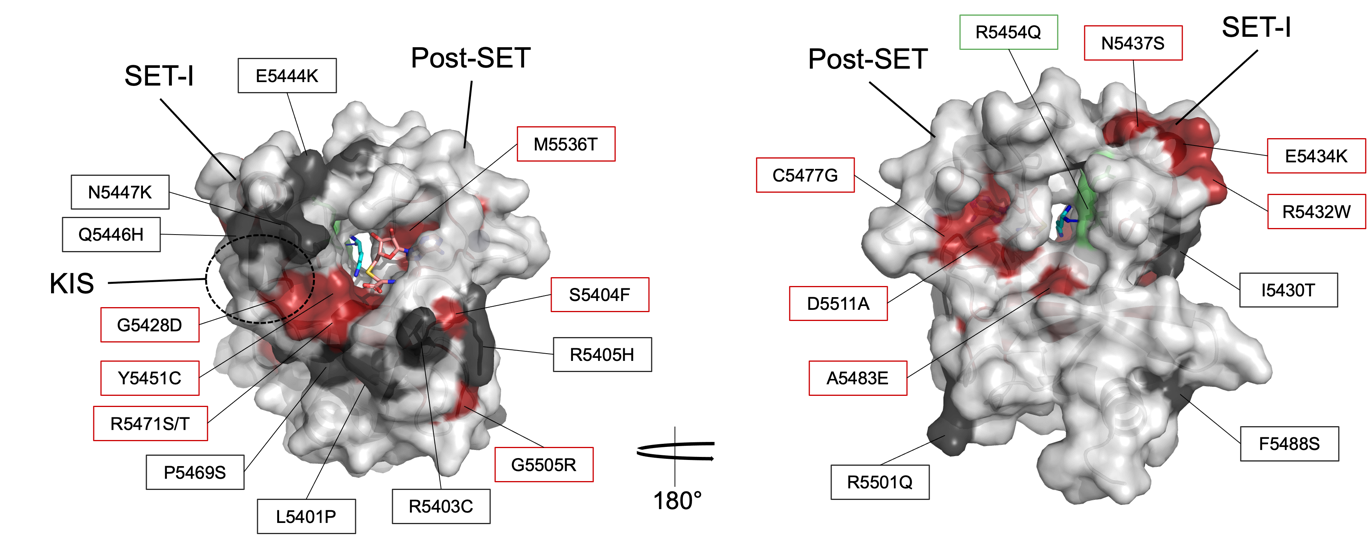


**B**

**Fig. S3**

**Fig. S4**


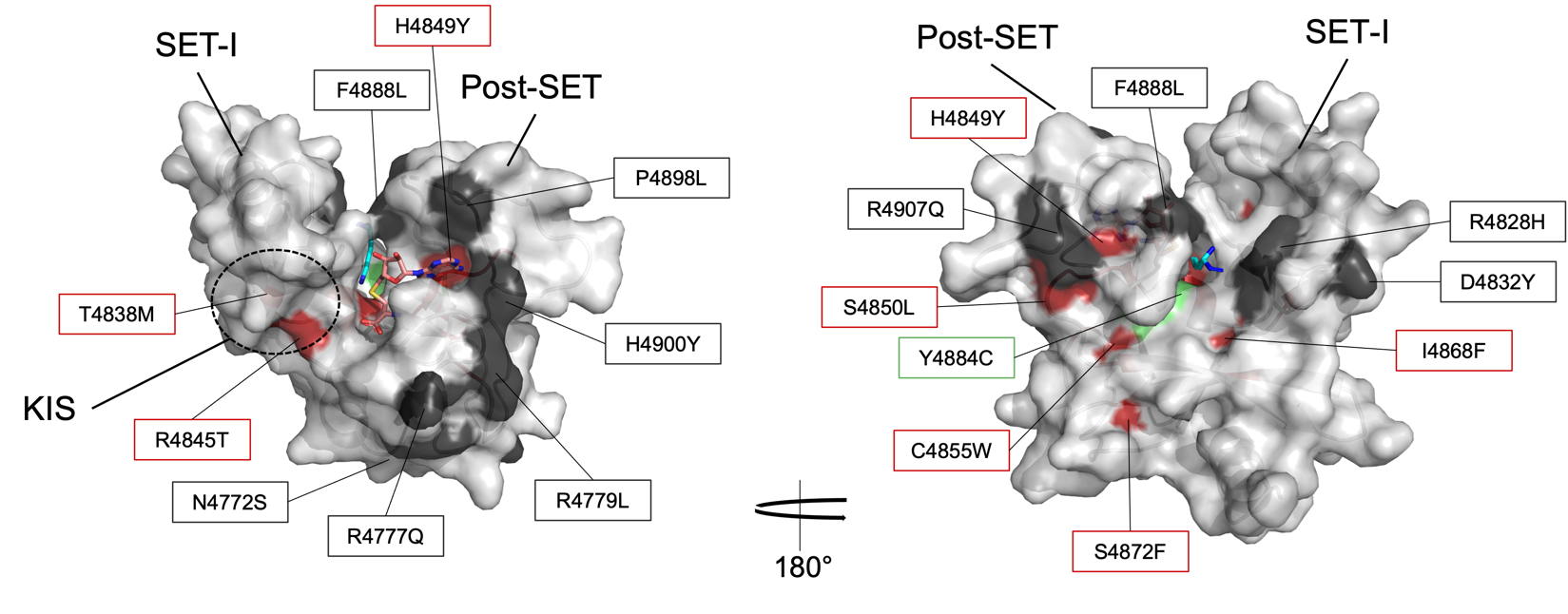


**B**

**Figure S4:** Parallel functional annotation assay results of MLL3 cancer-associated missense (CAM) mutations. (A) Top: (This figure is an adaptation of Fig. 2C and is shown here again for consistency with the previous annotations in Figs. S2 and S3.) MLL3 catalytic SET domain secondary structure map generated by PDBsum based on MLL3 structure (PDB 5F59, aa 4754-4911). Alpha helices (H1-3), beta sheets (β1-10) and respective beta hairpin turns (⊃) are annotated, and ligand/metal binding residues are labeled as follows: H3/SAH binding (▪️), SAH binding(▲), zinc ion binding (▪️/▲). Color coding: loss of mono- and dimethyltransferase activity (red), loss of only dimethylation activity (pink), gain of methyltransferase activity (green) or no change to catalytic activity (gray). Bottom, histogram showing results for assays initiated with H3K4me0 (unmethylated) and H3K4me1 (monomethylated) peptides, which are pink and purple bars, respectively. Horizontal dashed lines and corresponding shaded regions represent the average and one standard deviation (1σ), respectively, for all variants with activity greater than 50% of wild-type. Error bars represent standard deviations from 2 independent experiments. B) Results of MLL3 CAM mutations mapped onto the three-dimensional structure of the MLL3 SET domain (PDB code: (PDB 5F6K) (1) color coded as in (A).


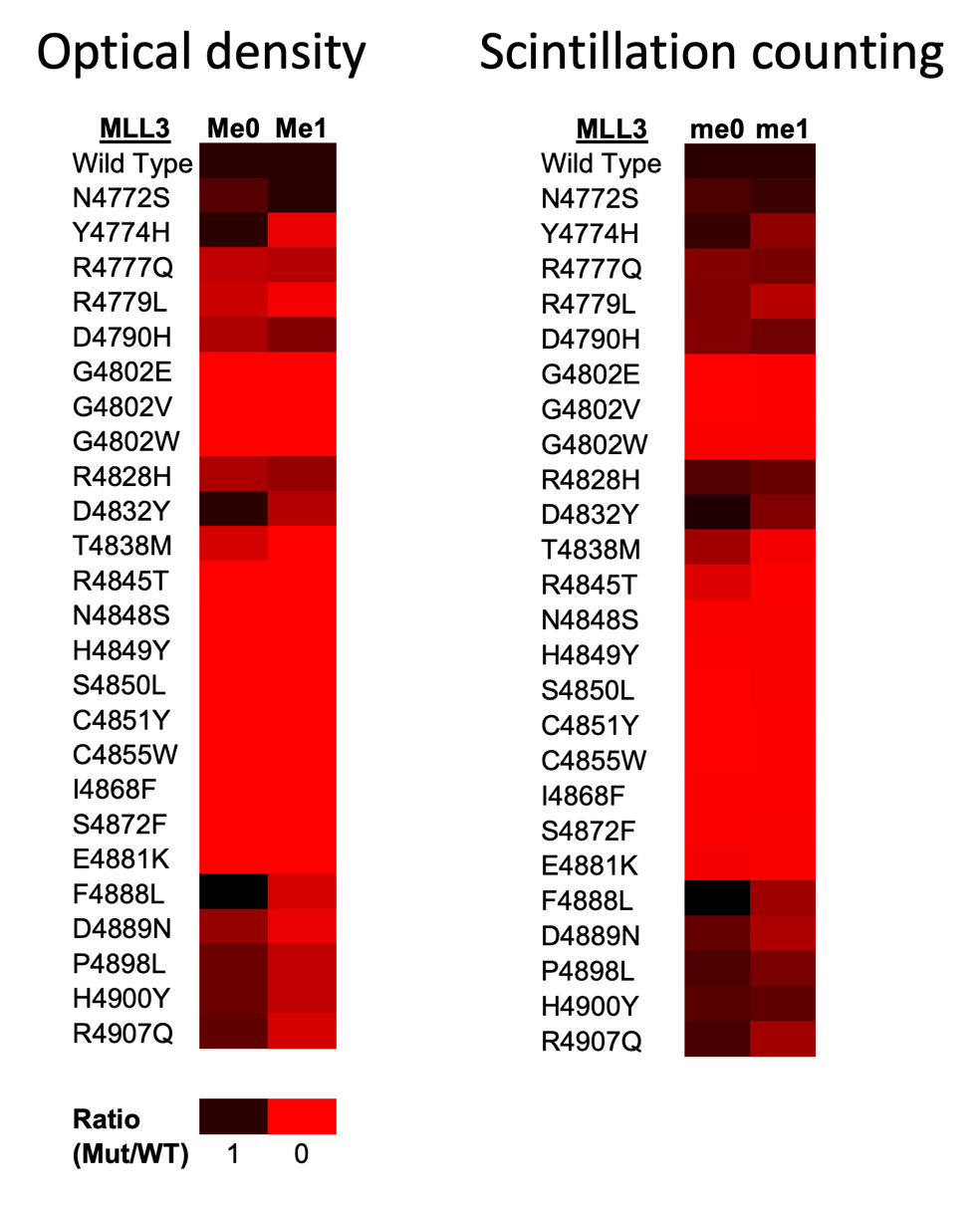


**Figure S5.** Comparison of parallel functional annotation assay results for gel-based fluorography and multiwell FlashPlate scintillation counting methods. Wild-type and mutant MLL3 variants were assayed as described in the text. Left, heat map based on optical density measurements from fluorographic films exposed for 6 hours. Right, heat map generated from scintillation counting. Results show good agreement, validating the use of 96-well FlashPlates for high-throughput assays.

**Fig. S5**

**Fig. S6**


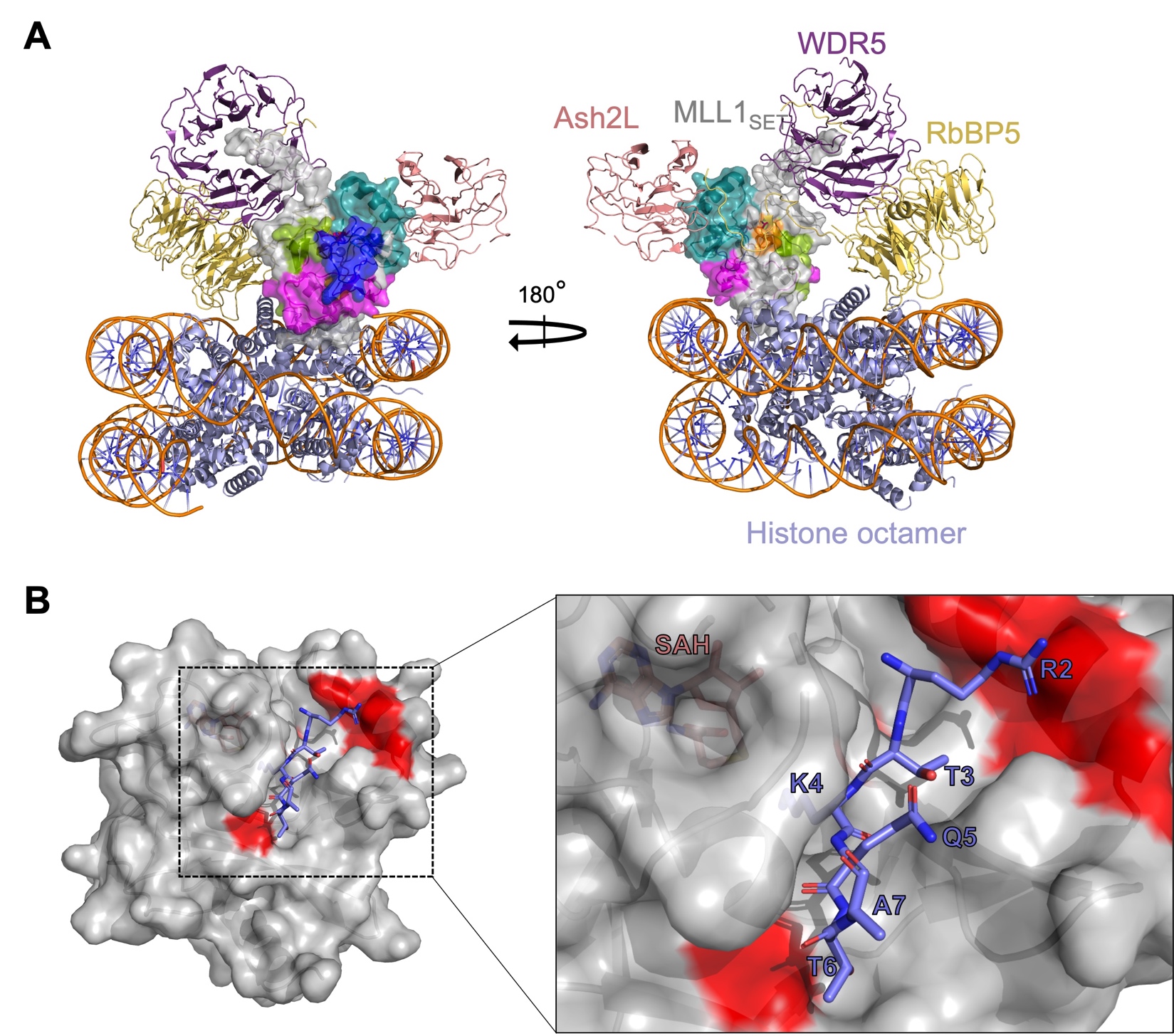


**Figure S6.** Substrate binding cleft mutation sites that result in a loss of function. (A) Loss of function mutation clusters of MLL1 (color coded as described in Results and Fig. 3B) within the MWRA complex bound to a nucleosome substrate. PDB: 6KIX. Histone proteins (H3, H4, H2A, H2B) in periwinkle, WDR5 in purple, RbBP5 in yellow, Ash2L in pink, and MLL1_SET_ base color in light grey. (B) Loss of function mutation positions from MLL1-3 mapped onto MLL3_SET_ structure (PDB 5F6K). LOF mutation sites shown in red sticks, Histone H3 peptide in blue, SAH in light pink.

**Fig. S7**

**Figure S7.** Missense mutation proximity scores were determined by projecting all missense mutations from MLL1, MLL2 and MLL3 onto a single SET sequence (*ProxRatioAll*) as described in Results and plotted as a function of MLL1 amino acid numbering.

**Fig. S8**

**Fig. S9**

**Figure S8.** Heat map of Spearman's correlation coefficients for the 14 potential explanatory variables and histone H3 lysine 4 methylation activity. Values are included in all squares with the exception of those that were zero.

**(A) Principal Components (Eigenvectors)**

| **Variable** | **PC1** | **PC2** | **PC3** | **PC4** |
| --- | --- | --- | --- | --- |
| ΔAtoms | 0.15 | -0.73 | 0.62 | -0.23 |
| ΔHBdon | 0.22 | -0.66 | -0.04 | 0.66 |
| ΔHBacc | -0.33 | 0.62 | 0.25 | 0.21 |
| ΔHydrophob | -0.26 | 0.20 | -0.06 | 0.75 |
| ΔCharge | 0.32 | -0.71 | -0.17 | 0.48 |
| ΔVol | 0.23 | -0.77 | -0.01 | -0.39 |
| Blosum62 | 0.33 | 0.18 | -0.35 | 0.15 |
| pClustScore | -0.57 | -0.09 | -0.81 | -0.06 |
| ProxRatioAll | -0.58 | -0.07 | -0.72 | -0.10 |
| ProxRatioEach | -0.47 | -0.07 | -0.77 | -0.02 |
| FI-score | -0.81 | -0.36 | 0.40 | -0.08 |
| VC-score | -0.77 | -0.38 | 0.40 | -0.10 |
| VS-score | -0.73 | -0.25 | 0.32 | -0.01 |
| ΔΔG | -0.48 | 0.05 | 0.29 | 0.57 |

**(B) Principal Component Regression Results**

| **Model** | | |  | |  | |  | |  | |  | | | | |  |  |  |  |  |
| --- | --- | --- | --- | --- | --- | --- | --- | --- | --- | --- | --- | --- | --- | --- | --- | --- | --- | --- | --- | --- |
| **Analysis of Variance** | | | **SS** | | **DF** | | **MS** | | **F (DFn, DFd)** | | | | | **P value** | |  |  |  |  |  |
| Regression | | | 7.8 | | 4 | | 2.0 | | F (4, 84) = 34 | | | | | P<0.0001 | |  |  |  |  |  |
| Residual | | | 4.9 | | 84 | | 0.058 | |  | | | | |  | |  |  |  |  |  |
| Total | | | 13 | | 88 | |  | |  | | | | |  | |  |  |  |  |  |
| **(C) Parameter Estimates** | | | |  | | | | | |  | |  |  |  | | | | |  |  |
| **Param.** | **Variable** | **Estimate** | | | | **Std. Err.** | | **95% CI (asymptotic)** | | | | | | **\|t\|** | **P value** | | **P value summary** |  |  |  |
| β0 | Intercept | 1.2 | | | | 0.063 | | 1.1 to 1.3 | | | | | | 19 | <0.0001 | | **** |  |  |  |
| β1 | ΔAtoms | -0.007 | | | | 0.003 | | -0.012 to -0.0013 | | | | | | 2.2 | 0.0033 | | ** |  |  |  |
| β2 | ΔHBdon | -0.004 | | | | 0.005 | | -0.017 to 0.0029 | | | | | | 1.4 | 0.4251 | | ns |  |  |  |
| β3 | ΔHBacc | -0.000 | | | | 0.003 | | -0.0077 to 0.0057 | | | | | | 0.1 | 0.9378 | | ns |  |  |  |
| β4 | ΔHydrophob | -0.001 | | | | 0.012 | | -0.039 to 0.0084 | | | | | | 1.3 | 0.4648 | | ns |  |  |  |
| β5 | ΔCharge | -0.004 | | | | 0.013 | | -0.037 to 0.015 | | | | | | 0.9 | 0.7365 | | ns |  |  |  |
| β6 | ΔVol | -0.000 | | | | 0.002 | | -0.00066 to 8.4e-005 | | | | | | 1.2 | 0.0545 | | ns |  |  |  |
| β7 | Blosum62 | 0.023 | | | | 0.003 | | 0.0095 to 0.023 | | | | | | 6.1 | <0.0001 | | **** |  |  |  |
| β8 | ClustScore | -0.009 | | | | 0.003 | | -0.018 to -0.0069 | | | | | | 4.7 | <0.0001 | | **** |  |  |  |
| β9 | ProxRatioAll | -0.014 | | | | 0.011 | | -0.02 to -0.009 | | | | | | 5.2 | <0.0001 | | **** |  |  |  |
| β10 | ProxRatioEach | -0.019 | | | | 0.005 | | -0.045 to -0.015 | | | | | | 4.3 | 0.0003 | | *** |  |  |  |
| β11 | FI-score | -0.060 | | | | 0.006 | | -0.070 to -0.048 | | | | | | 9.9 | <0.0001 | | **** |  |  |  |
| β12 | VC-score | -0.040 | | | | 0.004 | | -0.046 to -0.031 | | | | | | 9.9 | <0.0001 | | **** |  |  |  |
| β13 | VS-score | -0.075 | | | | 0.009 | | -0.092 to -0.062 | | | | | | 9.5 | <0.0001 | | **** |  |  |  |
| β14 | ΔΔG | -0.041 | | | | 0.010 | | -0.061 to -0.02 | | | | | | 3.9 | 0.0002 | | *** |  |  |  |
|  | | | |  | | | | | |  | |  |  |  | | | | |  |  |
| **(D) Goodness of Fit** | | | |  | | | | | |  | |  |  |  | | | | |  |  |
| Degrees of Freedom | | | | 84 | | | | | |  | |  |  |  | | | | |  |  |
| R squared | | | | 0.61 | | | | | |  | |  |  |  | | | | |  |  |
| Sum of Squares | | | | 4.9 | | | | | |  | |  |  |  | | | | |  |  |
| Sy.x | | | | 0.24 | | | | | |  | |  |  |  | | | | |  |  |

**Figure S9.** Initial principal component regression analysis of biophysical and sequence-based parameters impacting the variability in histone H3 lysine 4 methylation rates. (A) The overall variability in the data can be explained by the cumulative contributions of phylogenetic parameters (PC1), biophysical parameters (PC2), missense mutations clustering parameters (PC3) and hydrophobicity (PC4). Regression of these four principal components produced the results in panels (B-D).

**Fig. S10**

**(A) Principal Components (Eigenvectors)**

| **Variable** | **PC1** | **PC2** | **PC3** |
| --- | --- | --- | --- |
| ΔAtoms | 0.057 | -0.132 | 0.916 |
| Blosum62 | -0.359 | 0.371 | -0.207 |
| pClustScore | 0.589 | 0.800 | 0.036 |
| ProxRatioAll | 0.599 | 0.711 | 0.033 |
| ProxRatioEach | 0.491 | 0.766 | 0.038 |
| FI-score | 0.872 | -0.437 | 0.016 |
| VC-score | 0.840 | -0.439 | 0.083 |
| VS-score | 0.766 | -0.348 | -0.126 |
| *ΔΔG* | 0.416 | -0.279 | -0.415 |

**(B) Proportion of Variance**

| **Component** | **Eigenvalue** | **Proportion of variance** | **Cumulative proportion of variance** |
| --- | --- | --- | --- |
| 1 | 3.3 | 36.72% | 36.72% |
| 2 | 2.5 | 27.44% | 64.16% |
| 3 | 1.1 | 12.01% | 76.17% |

**(C) Principal Component Regression Results**

| **Model** |  |  |  |  |  |
| --- | --- | --- | --- | --- | --- |
| **Analysis of Variance** | **SS** | **DF** | **MS** | **F (DFn, DFd)** | **P value** |
| Regression | 7.8 | 3 | 2.6 | F (3, 85) = 44 | P<0.0001 |
| Residual | 5.0 | 85 | 0.058 |  |  |
| Total | 13.0 | 88 |  |  |  |

**(D) Parameter Estimates**

| **Param.** | **Variable** | **Estimate** | **Standard error** | **95% CI (asymptotic)** | **\|t\|** | **P value** | **P value summary** |
| --- | --- | --- | --- | --- | --- | --- | --- |
| β0 | Intercept | 1.153 | 0.065 | 1.043 to 1.297 | 18 | <0.0001 | **** |
| β1 | *ΔAtoms* | -0.014 | 0.007 | -0.028 to -0.002 | 2.1 | 0.0426 | * |
| β2 | *Blosum62* | 0.028 | 0.004 | 0.013 to 0.038 | 6.7 | <0.0001 | **** |
| β3 | *pClustScore* | -0.010 | 0.002 | -0.019 to -0.008 | 4.4 | <0.0001 | **** |
| β4 | *ProxRatioAll* | -0.014 | 0.003 | -0.128 to -0.078 | 5.0 | <0.0001 | **** |
| β5 | ProxRatioEach | -0.020 | 0.005 | -0.047 to -0.017 | 3.8 | 0.0003 | *** |
| β6 | *FI-score* | -0.060 | 0.006 | -0.069 to -0.046 | 10 | <0.0001 | **** |
| β7 | *VC-score* | -0.041 | 0.004 | -0.047 to -0.032 | 10 | <0.0001 | **** |
| β8 | *VS-score* | -0.070 | 0.008 | -0.085 to -0.048 | 8.9 | <0.0001 | **** |
| Β9 | *ΔΔG* | -0.026 | 0.012 | -0.050 to 0.00 | 2.2 | 0.0325 | * |

| **(E) Goodness of Fit** |  |
| --- | --- |
| Degrees of Freedom | 85 |
| R squared | 0.61 |
| Sum of Squares | 5.0 |
| Sy.x | 0.24 |

**Figure S10.** Principal component (PC) regression analysis of biophysical and sequence-based parameters impacting the variability in histone H3 lysine 4 methylation rates. (A) Table of eigenvectors with largest value for each parameter shaded in yellow. (B) The variability in the data can be explained by the cumulative contributions of phylogenetic parameters (PC1), clustering parameters (PC2), and amino acid physical-chemical properties (PC3). The results for the regression of these PCs on mutant activity relative to wild-type, *Activity^(Mut/WT)^*, are summarized in the panels (C-E).

**Fig. S11**

Cropped and Uncropped versions of Fig. 2C. Panels are arranged as in the Cropped version of Fig. 2C. The black boxes in the uncropped panels show the region of each that was used to create Fig. 2C.


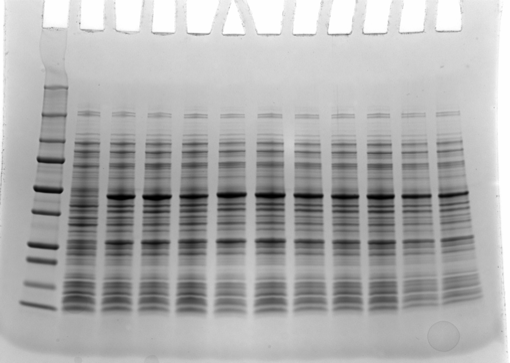

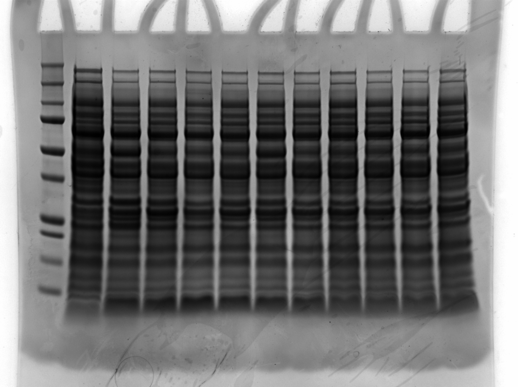

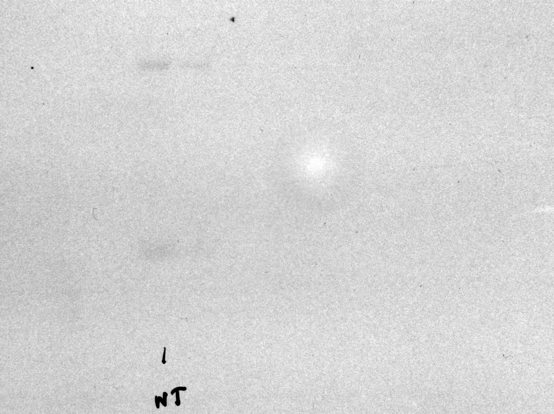

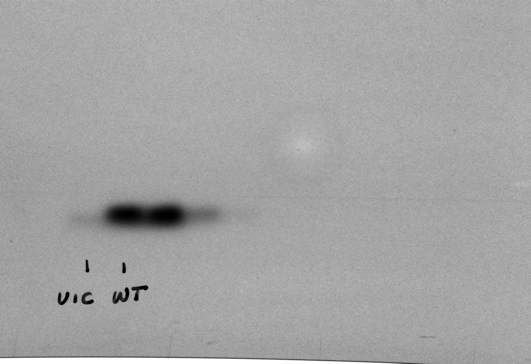

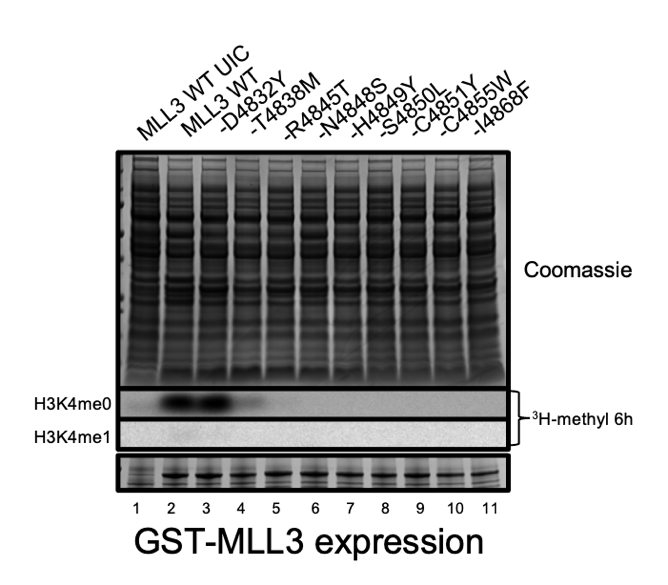


H3K4me1

^3^H-methyl 6h

Uncropped

Cropped

H3K4me0

^3^H-methyl 6h

Coomassie

Coomassie

**Table S1**

**Table S1**. Confusion matrix showing results from recursive partitioning with 10-fold crossvalidation (CV) as described in the text. TP, true positive; TN, true negative; FP, false positive; FN, false negative.

**Training Set**

|  | Prediction | |  |
| --- | --- | --- | --- |
| Actual | Positive | Negative | CV |
| Positive | 443 | 27 | 0.943 |
| Negative | 50 | 376 | 0.883 |
| Overall Accuracy= (TP+TN/TP+TN+FP+FN) = 0.914 | | | |

**Testing Set**

|  | | Prediction | | |  |
| --- | --- | --- | --- | --- | --- |
| Actual | | Positive | | Negative | CV |
| Positive | 48 | | 2 | | 0.96 |
| Negative | 5 | | 39 | | 0.89 |
|  | | | | | |
| Overall Accuracy: (TP+TN/TP+TN+FP+FN) = 0.93 | | | | | |

S

**Table S2**

**Table S2.** Comparison of functional inference parameters between this study and FATHMM and PolyPhen2 prediction programs.*

|  |  | **This Study** | **FATHMM** | **PolyPhen-2** | **CancerVar** |
| --- | --- | --- | --- | --- | --- |
| Sensitivity | TP/(TP+FN) | 0.91 | 0.06 | 1.00 | 0.63 |
| Specificity | TN/(TN+TP) | 0.93 | 0.98 | 1.00 | 0.61 |
| Precision | TP/(TP+FP) | 0.94 | 0.75 | 0.54 | 0.35 |
| Negative Predictive value | TN/(TN+FN) | 0.89 | 0.49 | 1.00 | 0.60 |
| Overall accuracy | (TP+TN)/(TP+TN+FP+FN) | 0.92 | 0.51 | 0.55 | 0.62 |

*Performance of each method was assessed as recommended by Vihinen (2012) (4)

TP = Number of True Positives

TN = Number True Negatives

FP = Number of False Positives

FN = Number of False Negatives

**Table S3**

**Table S3.** Least squares regression comparison models where *Activity^(Mut/WT)^* was regressed against *FI-Score* and either *pClustScore, ProxRatioAll, ProxRatioEach* or the combination of *ProxRatioAll* and *ProxRatioEach*. A) Akaike’s Information Criterion (AICc) was used to select the model that was most likely to have generated the data. B) Summary of fit statistics for each model listed in A.

| **Comparison of models** |  |  |  |
| --- | --- | --- | --- |
| Model 1 | *FI-Score* + *pClustScore* | *FI-Score* + *pClustScore* | *FI-Score + pClustScore* |
| Probability it is correct | 99.8% | 99.5% | 87.9% |
| Alternative model 2 | *FI-Score* + *ProxRatioAll* | *FI-Score* + *ProxRatioEach* | *FI-Score + ProxRatioALL + ProxRatioEach* |
| Probability it is correct | 0.231% | 0.539% | 12.1% |
| Ratio of probabilities | 433 | 184 | 7.24 |
| Preferred model | Model 1 | Model 1 | Model 1 |
| Difference in AICc | -12.1 | -10.4 | -3.96 |

| **Goodness of Fit** | ***pClustScore*** | ***ProxRatioAll*** | ***ProxRatioEach*** | ***ProxRatio(All+Each)*** |
| --- | --- | --- | --- | --- |
| DOF | 91 | 91 | 91 | 90 |
| Multiple R | 0.773 | 0.736 | 0.742 | 0.768 |
| R squared | 0.597 | 0.542 | 0.550 | 0.590 |
| Adjusted R squared | 0.589 | 0.532 | 0.540 | 0.576 |
| Sum of Squares | 5.45 | 6.21 | 6.09 | 5.55 |
| Sy.x | 0.245 | 0.261 | 0.259 | 0.248 |
| RMSE | 0.242 | 0.258 | 0.256 | 0.244 |
| AICc | -259 | -247 | -249 | -255 |

**References:**

1. Li, Y., Han, J., Zhang, Y., Cao, F., Liu, Z., Li, S., Wu, J., Hu, C., Wang, Y., Shuai, J., Chen, J., Cao, L., Li, D., Shi, P., Tian, C., Zhang, J., Dou, Y., Li, G., Chen, Y., and Lei, M. (2016) Structural basis for activity regulation of MLL family methyltransferases. *Nature* **530**, 447-452

2. Zhang, Y., Mittal, A., Reid, J., Reich, S., Gamblin, S. J., and Wilson, J. R. (2015) Evolving Catalytic Properties of the MLL Family SET Domain. *Structure* **23**, 1921-1933

3. Southall, S. M., Wong, P. S., Odho, Z., Roe, S. M., and Wilson, J. R. (2009) Structural basis for the requirement of additional factors for MLL1 SET domain activity and recognition of epigenetic marks. *Mol Cell* **33**, 181-191

4. Vihinen, M. (2012) How to evaluate performance of prediction methods? Measures and their interpretation in variation effect analysis. *BMC Genomics* **13 Suppl 4**, S2
